# Predicting major cardiac and cerebrovascular events in acute coronary syndrome patients using the thyroid hormone sensitivity index

**DOI:** 10.3389/fendo.2025.1543378

**Published:** 2025-07-02

**Authors:** Hui He, Jun Wang, Long Xia, Tao Ye, Yan Luo, Qiang Chen, Qiao Feng, Xiufen Peng, Maoling Jiang, Hanxiong Liu, Tao Xiang, Shiqiang Xiong, Lin Cai

**Affiliations:** ^1^ Department of Cardiology, The Affiliated Hospital, Southwest Medical University, Luzhou, Sichuan, China; ^2^ Department of Intensive Care Unit, Jinniu District People’s Hospital, Jinniu Hospital of Sichuan Provincial People’s Hospital, Chengdu, Sichuan, China; ^3^ Department of Cardiology, The Third People’s Hospital of Chengdu, Affiliated Hospital of Southwest Jiaotong University, Chengdu Cardiovascular Disease Research Institute, Chengdu, Sichuan, China; ^4^ Department of Emergency, The Third People’s Hospital of Chengdu, Affiliated Hospital of Southwest Jiaotong University, Chengdu, Sichuan, China

**Keywords:** acute coronary syndrome, percutaneous coronary intervention, thyroid hormone sensitivity, major adverse cardiac and cerebrovascular events, prognosis

## Abstract

**Background and aim:**

The thyroid hormone sensitivity index provides a new perspective for investigating nuanced alterations in thyroid function in cardiovascular disorders. However, the predictive value of thyroid hormone sensitivity indices for adverse events following percutaneous coronary intervention (PCI) in acute coronary syndrome (ACS) patients remains unknown. This study aimed to investigate the predictive value of thyroid sensitivity indices for major adverse cardiac and cerebrovascular events (MACCEs) in these patients.

**Methods and results:**

A total of 431 patients were included in the analysis. Thyroid hormone sensitivity indices were calculated using the thyroid-stimulating hormone index (TSHI), thyrotrophic thyroxine resistance index (TT4RI), thyroid feedback quantile-based index (TFQI), and parametric thyroid feedback quantile-based index (PTFQI). During the median follow-up period, 50 (11.60%) patients experienced MACCEs. Multivariate Cox regression analysis revealed that TSHI (HR 1.277, 95% CI 1.110–1.468, P<0.001), TT4RI (HR 1.002, 95% CI 1.001–1.003, P<0.001), TFQI (HR 1.130, 95% CI 1.043–1.224, P=0.003), and PTFQI (HR 1.237, 95% CI 1.107–1.383, P<0.001) were independent predictors of MACCEs. The area under the ROC curve (AUROC) revealed that PTFQI had the highest predictive value (AUROC =0.688, 95% CI: 0.595–0.780; P < 0.001). Adding PTFQI to the GRACE score can enhance the risk prediction of MACCEs in ACS patients undergoing PCI, AUROC = 0.759 (95% CI 0.676-0.842, P < 0.001) vs AUROC = 0.646 (95% CI 0.563-0.729, P = 0.001), and there is a significant difference (P = 0.0108). Subgroup analysis indicated that PTFQI had a more significant predictive value for MACCEs in males and patients with abnormal blood glucose.

**Conclusion:**

Thyroid hormone sensitivity indices are associated with a greater risk of MACCEs in patients with ACS following PCI.

## Introduction

Acute coronary syndrome (ACS) represents a considerable threat to global health, with a high prevalence of morbidity and mortality worldwide (1). Although interventions like reperfusion therapy, cardiac rehabilitation, and natural/herbal therapies have been shown to mitigate myocardial cell damage in myocardial infarction (MI), thereby improving outcomes in ACS patients (2–4). survivors of prior cardiovascular events still face a high risk of adverse outcomes. A study revealed that compared to non-MI individuals, those with post-circulatory disorder neuro-psychiatric diagnoses (e.g., anxiety, depression) exhibit more frequent MI trajectories and higher post-MI mortality (5). Given that late mortality in ACS patients correlates more with noncardiac factors (6). Research into biochemical indicators of coronary artery disease (CAD) has gained traction. For instance, one study identified distinct gut fungal dysbiosis across CAD progression, highlighting its potential as a biomarker and therapeutic target (7). Another study validated lncRNAs HCG15 and Morrbid as diagnostic/prognostic markers for acute myocardial infarction (AMI) (8). The significant physiological effect of thyroid hormones (THs) on the cardiovascular system has drawn attention to their potential role in predicting the clinical prognosis of CAD patients (9).

Previous studies have demonstrated that thyroid dysfunction (either excessive or deficient) is associated with the development or worsening of cardiovascular diseases, such as CAD, hypertension, and arrhythmias. The coordination of central negative feedback regulation, THs receptors in peripheral tissue, and deiodinases play vital roles in the homeostasis of THs (10). Therefore, a single serological index may not comprehensively reflect minor changes in thyroid function. The thyrotrophic thyroxine resistance index (TT4RI) (11) and thyroid-stimulating hormone index (TSHI) (12) were used to assess impaired central sensitivity to THs. In 2019, Laclaustra et al. proposed new indices for evaluating resistance to THs, including the thyroid feedback quantile-based index (TFQI) and the parametric thyroid feedback quantile-based index (PTFQI), and they reported a link between reduced central sensitivity to THs and the presence of diabetes and metabolic syndrome (13). The composite indices offer a more comprehensive understanding of subtle THs alterations and cardiovascular risks in the euthyroid population. Study has confirmed a negative correlation between the triglyceride-glucose (TyG) index (an indicator of insulin resistance) and central sensitivity to THs, evaluated using TSHI and TT4RI (14). A RCSCD-TCM study in China revealed a positive association between the TFQI, TTSHI, and TT4RI with an increased risk of carotid plaque development. Another search has shown that impaired sensitivity to THs (higher quartiles of the TFQI, PTFQI, TSHI, and TT4RI) is associated with elevated homocysteine (Hcy) levels in the euthyroid population (15). The studies suggest that reduced sensitivity to THs is significantly linked to multiple high-risk factors that contribute to the suppression of CAD. However, the prognostic significance of these novel indices of THs resistance for adverse outcomes in ACS patients undergoing percutaneous coronary intervention (PCI) remains unexplored.

Therefore, the objective of this study was to assess the predictive value of thyroid hormone sensitivity for major adverse cardiac and cerebrovascular events (MACCEs) in ACS patients undergoing PCI.

## Methods

### Enrolled population

In this single-center observational research study, a total of 1827 consecutive patients diagnosed with ACS and underwent PCI at the Third People’s Hospital of Chengdu (Sichuan, China) from July 2018 to December 2020 were enrolled. Participants were excluded if they (1) had valvular disease necessitating cardiac surgery; (2) had a prior history of coronary artery bypass grafting(CABG); (3) presented severe hepatic and renal insufficiency with creatinine clearance <15 ml/min; (4) had hypophysoma or other malignant tumors; (5) Individuals taking medications that affect thyroid function (such as anti-thyroid drugs, glucocorticoids, iodine-containing drugs, dopamine agonists, etc.); or (6) lacked essential variables, such as thyroid hormone and coronary angiography data.

**Figure 1:**
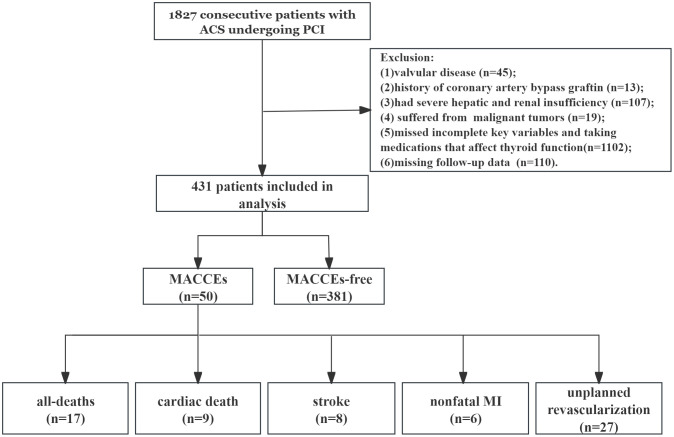
Flow chart of the study population. ACS, acute coronary syndrome; PCI, percutaneous coronary intervention; MACCEs, major adverse cardiac and cerebrovascular events; MI, myocardial infarction.

Patients missing follow-up data despite at least three contact attempts were also excluded. Ultimately, 431 patients were enrolled in the final analyses (**Figure 1**).

The study protocol was approved by the local research ethics committee and strictly complied with the Declaration of Helsinki. Personal information related to the identities of the patients was concealed.

### Anthropometric and biochemical measurements

Demographics (sex, age), anthropometric data, clinical diagnoses (diabetes mellitus, hypertension, heart failure, etc.), symptoms and clinical signs at admission, laboratory examinations, echocardiographic results, records of PCI surgery, and medication at hospital discharge were collected. Body mass index (BMI) was calculated as follows: BMI=weight (kg)/[height (m)]^2^.

Peripheral venous blood samples were obtained after at least 8h of overnight fasting. The concentrations of THs, cardiac troponin T (cTnT), brain natriuretic peptide (BNP), total cholesterol (TC), triglycerides (TGs), low-density lipoprotein-C (LDL-C), high-density lipoprotein-C (HDL-C), albumin and fibrinogen were determined using standard biochemical techniques. The formula for calculating the creatinine clearance rate (Ccr) in males is (140 - age) * weight (kg)/72 * serum creatinine (mg/dl), and for females, the result is multiplied by 0.85.

### Thyroxines, TSH, and thyroid hormone sensitivity indices

The reference ranges of thyroid function were 2.63–5.70 pmol/L for FT3, 9.01–19.05 pmol/L for FT4, and 0.35–4.94 mU/L for TSH. The thyroid hormone sensitivity indices, including the TT4RI, TSHI, TFQI, and PTFQI, were evaluated based on the composite index of TSH and FT4, which reflects the sensitivity of the hypothalamic-pituitary-thyroid (HPT) axis to changes in circulating FT4 concentrations. Elevated of the thyroid hormone sensitivity indices levels indicate decreased sensitivity to THs.

The thyroid hormone sensitivity indices were calculated using the following formula:

TSHI=lnTSH(mU/L)+0.1345×FT4(pmol/L) (12),

TT4RI=FT4(pmol/L)×TSH(mU/L) (11),

TFQI=cdf FT4−(1−cdf TSH) (13),

PTFQI=Φ((FT4−μFT4)/σFT4)−(1−Φ((ln TSH−μln TSH)/σln TSH)) (13). In this population, µFT4 was 12.8409, σFT4 was 0.5579, µLn TSH was 0.5811, and σLn TSH was 0.8141.

### Follow-up and endpoints

Clinical follow-up was scheduled at 3, 6, and 12 months and then annually after hospital discharge via clinical visits or telephone questionnaires. The primary endpoint was a composite of MACCEs, including all-cause death, nonfatal MI, stroke, and unplanned revascularization during follow-up. The secondary endpoints included all-cause death, cardiac death, nonfatal MI, nonfatal stroke, and unplanned revascularization. All clinical endpoints were documented and verified by referring to relevant medical records when available. All-cause death referred to death regardless of the cause. Cardiac deaths were considered cardiac unless an unequivocal noncardiac cause could be established. Stroke was defined as an ischemic or hemorrhagic stroke that occurred during the follow-up period (confirmed by neurological dysfunction or clinically documented lesions on imaging). Nonfatal MI was defined as elevated creatine kinase or cardiac troponin levels greater than the upper limit of the normal range, with ischemia indicated by electrocardiographic changes or symptoms. Unplanned revascularization was defined as an ischemia-driven target or nontarget revascularization after the index PCI or CABG during the follow-up period.

### Statistical analysis

Normality was examined via the Kolmogorov–Smirnov test. Normally distributed variables are presented as means ± standard deviations, whereas skewed variables are presented as medians (interquartile ranges, IQRs). Data for categorical variables are expressed as percentages (%). The study participants were stratified into two groups based on the primary endpoint. We used the independent samples t test or the Mann–Whitney U test for continuous variables and the chi-square test for categorical variables to determine differences in baseline characteristics among groups. Thyroid hormone sensitivity indices were analyzed as continuous variables. A receiver operating characteristic (ROC) curve was constructed to determine the diagnostic accuracy of the thyroid hormone sensitivity indices. And we complemented the statistical approach with FDR correction analysis.

We chose the multivariate Cox proportional hazards regression model to correct for multiple confounding factors, as it is well-suited for analyzing time-to-event data and enables adjustment for confounding factors to estimate independent hazard ratios. And we assessed the proportional hazards assumption of the Cox model through methods such as the Schoenfeld residual test and did not detect any substantial breaches. Furthermore, Kaplan–Meier survival analysis was performed to evaluate the relationships between tertiles of the best predictive index and the cumulative incidence of MACCEs. The log-rank test was used to compare the differences between groups. All tests were two-tailed, and P <0.05 was considered statistically significant.

All statistical analyses were conducted using IBM SPSS Statistics 29.0.1.0 software (IBM Corporation, Armonk, NY, USA), GraphPad Prism 10.1.2, and MedCalc^®^ statistical software.

## Results

### Baseline characteristics of the patients stratified by the primary endpoint

The cohort study’s median follow-up period was 35 (IQR, 29–41) months. The baseline characteristics of the total population are presented in **Table 1**. Among all patients, the median age was 69 (IQR, 60-77) years, with 74.0% male and 11.6% experiencing MACCEs. Patients who experienced MACCEs were more likely to be male and less likely to be current smokers. The levels of age, urea nitrogen, fasting blood glucose, fibrinogen, TSH, and thyroid hormone sensitivity indices were significantly higher in patients with MACCEs, had higher rates of diabetes mellitus. There were no significant differences between the groups in terms of diagnosis (unstable angina, STEMI, and NSTEMI). Moreover, regarding coronary procedural information, **Table 2** shows that patients with MACCEs presented higher rates of multivessel disease (MVD) and coronary rotablation, a greater number of stents implanted, longer stent lengths, and were more likely to be discharged on diuretics and insulin therapy.

**Table 1:** Baseline characteristics of the patients stratified by the primary endpoint.

Varies	Total (n=431)	MACCEs (n=50)	MACCEs-free (n=381)	P value
Patient characters				
Male, n (%)	319 (74.0)	31 (62.0)	288 (75.6)	**0.039**
Age, years	69 (60, 77)	75 (63, 80)	68 (59, 76)	**0.027**
BMI, kg/m2	24.66 ± 3.29	24.82 ± 3.23	24.64 ± 3.30	0.973
Current smoking, n (%)	163 (37.82)	10 (20.0)	153 (40.2)	**0.006**
Alcohol, n (%)	152 (35.30)	14 (28.0)	138 (36.2)	0.253
Previous History				
Hypertension, n (%)	299 (69.4)	37 (74.0)	262 (68.8)	0.451
Diabetes mellitus, n (%)	114 (26.45)	22 (44.0)	92 (24.1)	**0.003**
Previous CAD, n (%)	82 (19)	13 (26.0)	69 (18.1)	0.182
Previous PCI, n (%)	30 (7)	4 (8.0)	26 (6.8)	0.759
Previous COPD, n (%)	16 (3.7)	2 (4.0)	14 (3.7)	0.909
Atrial fibrillation, n (%)	14 (3.2)	0 (0)	14 (3.7)	0.169
Stroke, n (%)	23 (5.3)	3 (6.0)	20 (5.2)	0.824
Chronic kidney diseases, n (%)	8 (1.9)	0 (0)	8 (2.1)	0.301
Diagnosis, n (%)				
UA, n (%)	212 (49.2)	25 (50.0)	187 (49.1)	0.903
STEMI, n (%)	104 (24.1)	17 (37.0)	87 (22.8)	0.083
NSTEMI, n (%)	115 (26.7)	8 (16.0)	107 (28.1)	0.070
SBP (mmHg)	133 (120, 148)	133 (120, 146)	133 (120, 149)	0.955
DBP (mmHg)	77 (70, 88)	76 (69, 84)	77 (70, 89)	0.276
Heart rate (bpm)	77 (67, 84)	77 (67, 86)	77 (67, 84)	0.787
Troponin-T (pg/ml)	27.1 (10.6, 290.4)	30.8 (13.1, 92.7)	27.0 (10.5, 347.2)	0.877
BNP (pg/ml)	104.1 (41.3, 274.7)	120.2 (49.4, 325.1)	100.4 (39.2, 275.5)	0.366
Ccr (ml/min)	71.09 (54.25, 89.91)	66.43 (50.83, 78.45)	71.8 (54.69, 91.62)	0.100
Urea nitrogen (μmol/l)	5.68 (4.53, 7.16)	6.55 (5.30, 7.84)	5.62 (4.46, 7.1)	**0.004**
Fasting blood-glucose (mmol/l)	5.74 (5.01, 7.37)	6.54 (5.42, 8.31)	5.59 (4.98, 7.29)	**0.004**
Triglyceride (mmol/l)	1.47 (1.06, 2.03)	1.44 (3.55, 5.14)	1.47 (1.04, 2.01)	0.510
Total cholesterol (mmol/l)	4.35 (3.51, 5.12)	4.49 (3.54, 5.14)	4.33 (3.51, 5.11)	0.575
LDL-C (mmol/l)	2.62 (2.01, 3.27)	2.72 (1.89, 3.30)	2.61 (2.01, 3.27)	0.987
HDL-C (mmol/l)	1.11 (0.95, 1.33)	1.14 (0.99, 1.39)	1.1 (0.95, 1.32)	0.574
Albumin (g/l)	39.9 (36.9, 42.4)	40.2 (36.7, 42.1)	39.9 (36.9, 42.5)	0.990
Fibrinogen (g/l)	3.59 (3.03, 4.51)	4.29 (3.50, 5.03)	3.50 (2.98, 4.37)	**<0.001**
TSH (mIU/L)	1.90 (1.13, 3.05)	3.28 (2.04, 4.66)	1.75 (1.09, 2.75)	**<0.001**
FT3 (pmol/L)	3.77 (3.31, 4.20)	3.70 (3.13, 4.19)	3.79 (3.34, 4.21)	0.403
FT4 (pmol/L)	12.5 (11.4, 13.7)	12.8 (10.9, 14.7)	12.5 (11.4, 13.6)	0.261
TSHI	2.29 (1.76, 2.76)	2.89 (2.15, 3.41)	2.25 (1.74, 2.70)	**<0.001**
TT4RI	22.57 (13.57, 36.54)	40.02 (21.77, 59.43)	21.77 (13.30, 34.34)	**<0.001**
TFQI	0.00 ± 0.38	0.21 ± 0.46	-0.02 ± 0.36	**<0.001**
PTFQI	0.00 (-0.24, 0.20)	0.24 (-0.06, 0.39)	-0.03 (-0.26, 0.17)	**<0.001**
LVEF (%)	57 (50, 61)	59 (51, 62)	58 (53, 63)	0.576

The patients were divided into two groups based on the primary endpoint. Data are presented as the mean ± SD, median (upper and lower quartiles) or number (%). Bold indicates P value < 0.05. MACCEs,major adverse cardiac and cerebrovascular events; BMI, body mass index; CAD, coronary artery disease; PCI, percutaneous coronary intervention; COPD, chronic obstructive pulmonary disease; UA, unstable angina; STEMI, ST-segment elevation myocardial infarction; NSTEMI, non-ST-segment elevation myocardial infarction; SBP, systolic blood pressure; DBP, diastolic blood pressure; BNP, brain natriuretic peptide; Ccr, Creatinine clearance rate; LDL-C, low-density lipoprotein cholesterol; HDL-C, high-density lipoprotein cholesterol; TSH, thyroid-stimulating hormone; FT3, free triiodothyronine; FT4, free thyroxine; TSHI, thyroid-stimulating hormone index; TT4RI, thyrotrophic thyroxine resistance index; TFQI, thyroid feedback quantile-based index; PTFQI, and parametric thyroid feedback quantile-based index; LVEF, left ventricular ejection fraction.

**Table 2:** Angiographic data and discharge medications of the patients stratified by the primary endpoint.

Varies	Total (n=431)	MACCEs (n=50)	MACCEs-free (n=381)	P value
Angiographic data				
MVD, n (%)	136 (31.6)	23 (46.0)	113 (29.7)	**0.020**
LM, n (%)	21 (4.9)	3 (6.0)	18 (4.7)	0.820
Branches lesions, n (%)	26 (6.0)	4 (8.0)	22 (5.8)	0.918
Calcified lesions, n (%)	59 (13.7)	13 (26.0)	46 (12.1)	0.275
Thrombosis, n (%)	20 (4.6)	2 (4.0)	18 (4.7)	0.472
CTO, n (%)	73 (16.9)	10 (20.0)	63 (16.5)	0.849
Long lesion, n (%)	136 (31.6)	22 (44.0)	114 (29.9)	0.287
Coronary rotablation, n (%)	7 (1.6)	3 (6.0)	4 (1.0)	**0.009**
Thrombus aspiration, n (%)	8 (1.9)	0 (0)	8 (2.1)	0.301
Number of stents	1 (1, 2)	1 (1, 2)	1 (1, 2)	**0.035**
Length of stents (mm)	29 (20, 48)	37 (23, 61)	29 (20, 46)	**0.045**
Discharge medications				
Aspirin, n (%)	420 (97.4)	48 (96.0)	372 (97.6)	0.490
Clopidogrel, n (%)	425 (98.6)	49 (98.0)	376 (98.7)	0.697
Anticoagulation, n (%)	15 (3.5)	2 (4.0)	13 (3.4)	0.831
Statins, n (%)	421 (97.7)	48 (96.0)	273 (97.9)	0.402
Diuretics, n (%)	69 (16)	13 (26.0)	56 (14.7)	**0.041**
β-blocker, n (%)	294 (68.2)	32 (64.0)	262 (68.8)	0.497
ACEI/ARB, n (%)	214 (49.7)	28 (56.0)	186 (48.8)	0.340
Insulin treatment, n (%)	32 (7.4)	9 (18.0)	23 (6)	**0.002**
Hypoglycemic agent, n (%)	95 (22)	18 (36.0)	77 (20.2)	**0.011**

The patients were divided into two groups based on the primary endpoint. Data are presented as median (upper and lower quartiles) or number (%). Bold indicates P value < 0.05. MACCEs, major adverse cardiac and cerebrovascular events; MVD, multivessel disease; LM, left main disease; CTO, chronic total occlusion; ACEI/ARB, angiotensin converting enzyme inhibitor/angiotensin receptor blocker.

### Association between the thyroid hormone sensitivity indices and MACCEs

To further investigate the potential relationship between thyroid hormone sensitivity and MACCEs, Cox regression analysis was conducted on all patients. Univariate Cox regression analysis identified sex, age, smoking, diabetes, BNP, Ccr, TSH, FT4, TSHI, TT4RI, TFQI, PTFQI, MVD, diuretics, and insulin as significant risk factors for MACCEs following PCI in patients with ACS (all P<0.05, to avoid mutual interference between the thyroid hormone sensitivity indices, separate tables were created for these variables (refer to the **Supplementary Tables 1-4**). After adjusting for potential confounders, multivariate Cox regression analysis revealed that as continuous variables, TSHI (HR 1.277, 95% CI 1.110–1.468, P<0.001), TT4RI (HR 1.002, 95% CI 1.001–1.003, P<0.001), TFQI (HR 1.130, 95% CI 1.043–1.224, P=0.003) and PTFQI (HR 1.237, 95% CI 1.107–1.383, P<0.001) were positively correlated with the risk of MACCEs in ACS patients following PCI. As TT4RI: HR = 1.002, suggests that an elevated TT4RI shows minimal linear correlation with the risk of MACCEs. We further investigated the relationships between thyroid hormone sensitivity indices by categorizing them as tertiles. When T1 was set as the reference, the HR for MACCEs increased significantly with the highest tertile of TSHI (HR 3.722, 95% CI 1.468–9.438, P=0.006), TT4RI (HR 3.909, 95% CI 1.557–9.813, P=0.004), TFQI (HR 3.792, 95% CI 1.470–9.783, P=0.006), and PTFQI (HR 3.684, 95% CI 1.374–9.875, P=0.010). This finding underscores a notable association between the risk of MACCEs and thyroid hormone sensitivity indices. While the risk remains relatively unchanged in the moderate to low levels (T1-T2), possibly due to the insufficient sample size leading to the nonsignificant differential effect in the moderate to low groups (**Table 3**).

**Table 3:** COX regression analysis for the association of the tertiles of the thyroid hormone sensitivity indices and MACCEs.

Variables	Model 1		Model 2		Model 3	
	HR (95% CI)	Pvalue	HR (95% CI)	P value	HR (95% CI)	P value
TSHI	1.305 (1.142-1.491)	**<0.001**	1.312 (1.144-1.504)	**<0.001**	1.277 (1.110-1.468)	**<0.001**
T1 (<2.01)	Reference		Reference		Reference	
T2 (2.01 to 2.62)	1.127 (0.458-2.777)	0.794	1.099 (0.443-2.726)	0.839	2.017 (0.717-5.676)	0.184
T3 (>2.62)	2.887 (1.324-6.297)	**0.008**	2.662 (1.209-5.859)	**0.015**	3.722 (1.468-9.438)	**0.006**
TT4RI	1.001 (1.001-1.002)	**<0.001**	1.001 (1.001-1.002)	**<0.001**	1.002 (1.001-1.003)	**<0.001**
T1 (<17.27)	Reference		Reference		Reference	
T2 (1.27 To 31.77)	0.857 (0.329-2.233)	0.752	0.765 (0.290-2.019)	0.589	1.320 (0.439-3.970)	0.621
T3 (>31.77)	3.181 (1.464-6.910)	**0.003**	2.990 (1.356-6.359)	**0.007**	3.909 (1.557-9.813)	**0.004**
TFQI	1.253 (1.119-1.403)	**<0.001**	1.249 (1.112-1.402)	**<0.001**	1.130 (1.043-1.224)	**0.003**
T1 (<-0.14)	Reference		Reference		Reference	
T2 (-0.14 to 0.15)	1.817 (0.726-4.551)	0.202	1.897 (0.757-4.756)	0.172	2.671 (0.985-7.241)	0.053
T3 (>0.15)	3.136 (1.338-7.354)	**0.009**	3.065 (1.303-7.207)	**0.010**	3.792 (1.470-9.783)	**0.006**
PTFQI	1.235 (1.101-1.387)	**<0.001**	1.243 (1.105-1.398)	**<0.001**	1.237 (1.107-1.383)	**<0.001**
T1 (<-0.14)	Reference		Reference		Reference	
T2 (-0.14 to 0.14)	1.035 (0.418-2.562)	0.941	1.057 (0.426-2.621)	0.904	2.113 (0.727-6.146)	0.169
T3 (>0.14)	2.300 (1.053-5.023)	**0.037**	2.319 (1.066-5.047)	**0.034**	3.684 (1.374-9.875)	**0.010**

The patients were divided into three groups in accordance with tertiles of the thyroid hormone sensitivity indices. Data were expressed as odds ratio (OR) and 95% confidence interval (CI). Bold indicates P value < 0.05.

Model 1: adjusted for age and sex.

Model 2: adjusted for age, sex, Smoking, diabetes.

Model 3: adjusted for age, sex, smoking, diabetes, BNP, fibrinogen, Ccr, MVD, diuretics and insulin treatment. BNP, brain natriuretic peptide; Ccr, Creatinine clearance rate, MVD, multivessel disease.

**Figure 2:**
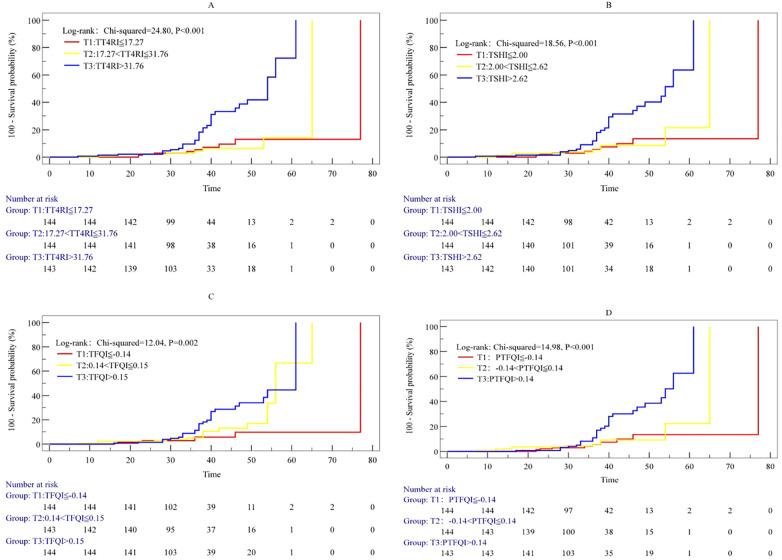
Cumulative incidence of the primary endpoint events according to the tertiles of thyroid hormone sensitivity indices. The groups were stratified by the tertiles of thyroid hormone sensitivity indices. Kaplan–Meier curves for the incidence of the primary endpoint among the 3 study groups based on TT4RI **(A)**, TSHI **(B)**, TFQI **(C)**, and PTFQI **(D)**. TT4RI, thyrotrophic thyroxine resistance index; TSHI, thyroid-stimulating hormone index; TFQI, thyroid feedback quantile-based index; PTFQI, parametric thyroid feedback quantile-based index.

In the overall population, Kaplan-Meier curve log-rank tests revealed that TT4RI (Chi-squared=24.80, P<0.001), TSHI (Chi-squared=18.56, P<0.001), TFQI (Chi-squared=12.04, P=0.002), and PTFQI (Chi-squared=14.98, P<0.001) all showed statistically significant differences in survival time for MACCEs (**Figure 2**).

### Diagnostic performance of thyroid hormone sensitivity for the primary endpoint

**Figure 3:**
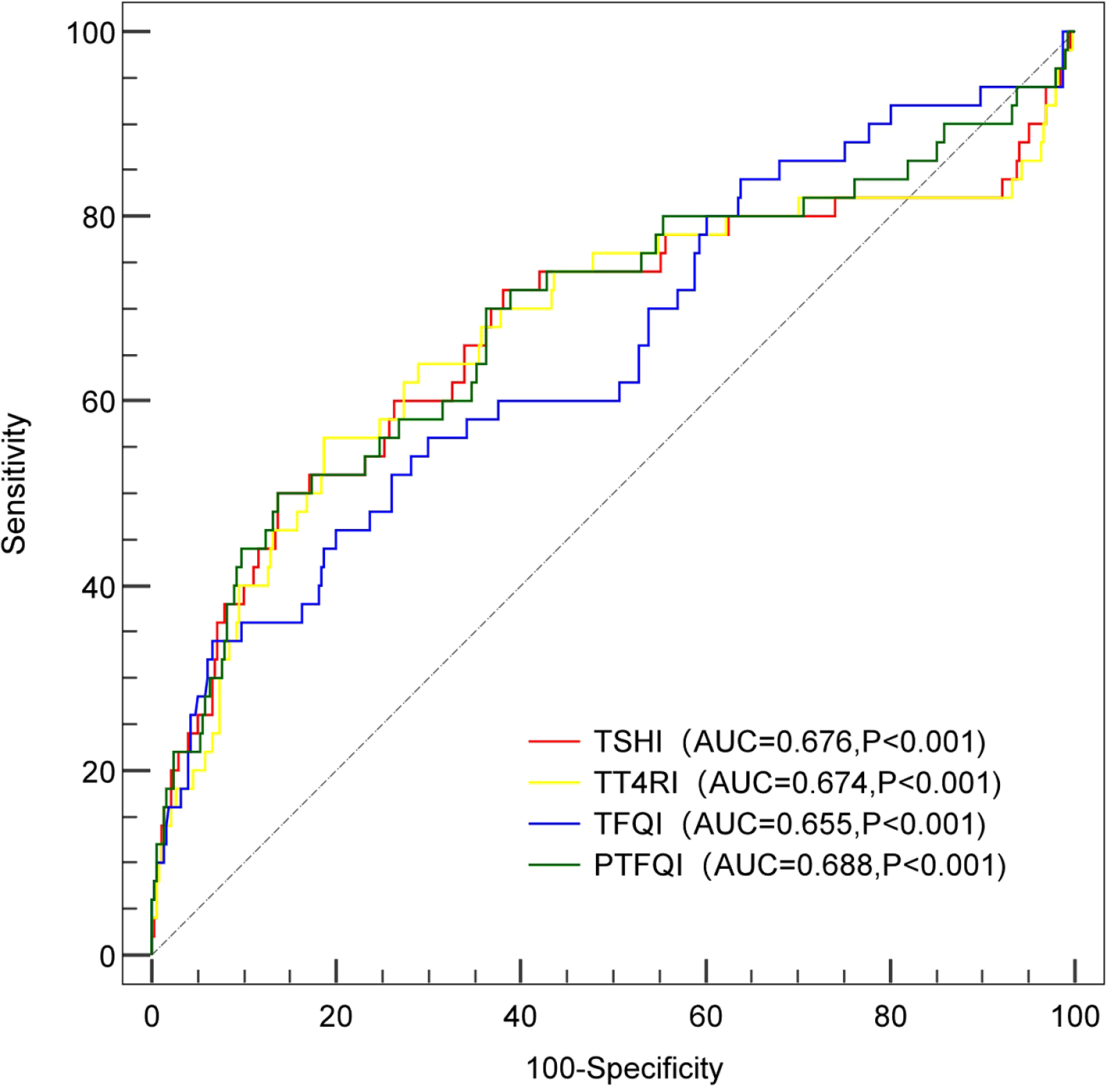
ROC curve analysis of thyroid hormone sensitivity indices for MACCEs. The area under the ROC curve (AUROC) of for thyroid hormone sensitivity indices predicting the occurrence of MACCEs. TSHI was 0.676 (95% CI: 0.578 - 0.774; P<0.001); TT4RI was 0.674 (95% CI: 0.577 - 0.771; P<0.001); TFQI was 0.655 (95% CI: 0.566 - 0.744; P<0.001); PTFQI was 0.688 (95% CI: 0.595 - 0.780; P<0.001). MACCEs, major adverse cardiac and cerebrovascular events; ROC, receiver operating characteristic; PCI, percutaneous coronary intervention. TSHI, thyroid-stimulating hormone index; TT4RI, thyrotrophic thyroxine resistance index; TFQI, thyroid feedback quantile-based index; PTFQI, and parametric thyroid feedback quantile-based index.

The predictive value of the thyroid hormone sensitivity indices for MACCEs was estimated using ROC curves. The results indicate that the optimal cutoff value for PTFQI is 0.26, with an area under the ROC curve (AUROC) of 0.688 (95% CI: 0.595–0.780; P<0.001). The TSHI was 2.94, with an AUROC of 0.676 (95% CI: 0.578–0.774; P<0.001). The TT4RI was 38.61, with an AUROC of 0.674 (95% CI: 0.577–0.771; P<0.001). The TFQI was 0.55, with an AUROC of 0.655 (95% CI: 0.566–0.744; P<0.001) (**Figure 3**). TT4RI, TSHI, TFQI, and PTFQI all have moderate predictive abilities for MACCEs in ACS patients after PCI, and PTFQI exhibited the highest accuracy.

### Additional predictive values of PTFQI in the GRACE risk prediction model

**Figure 4:**
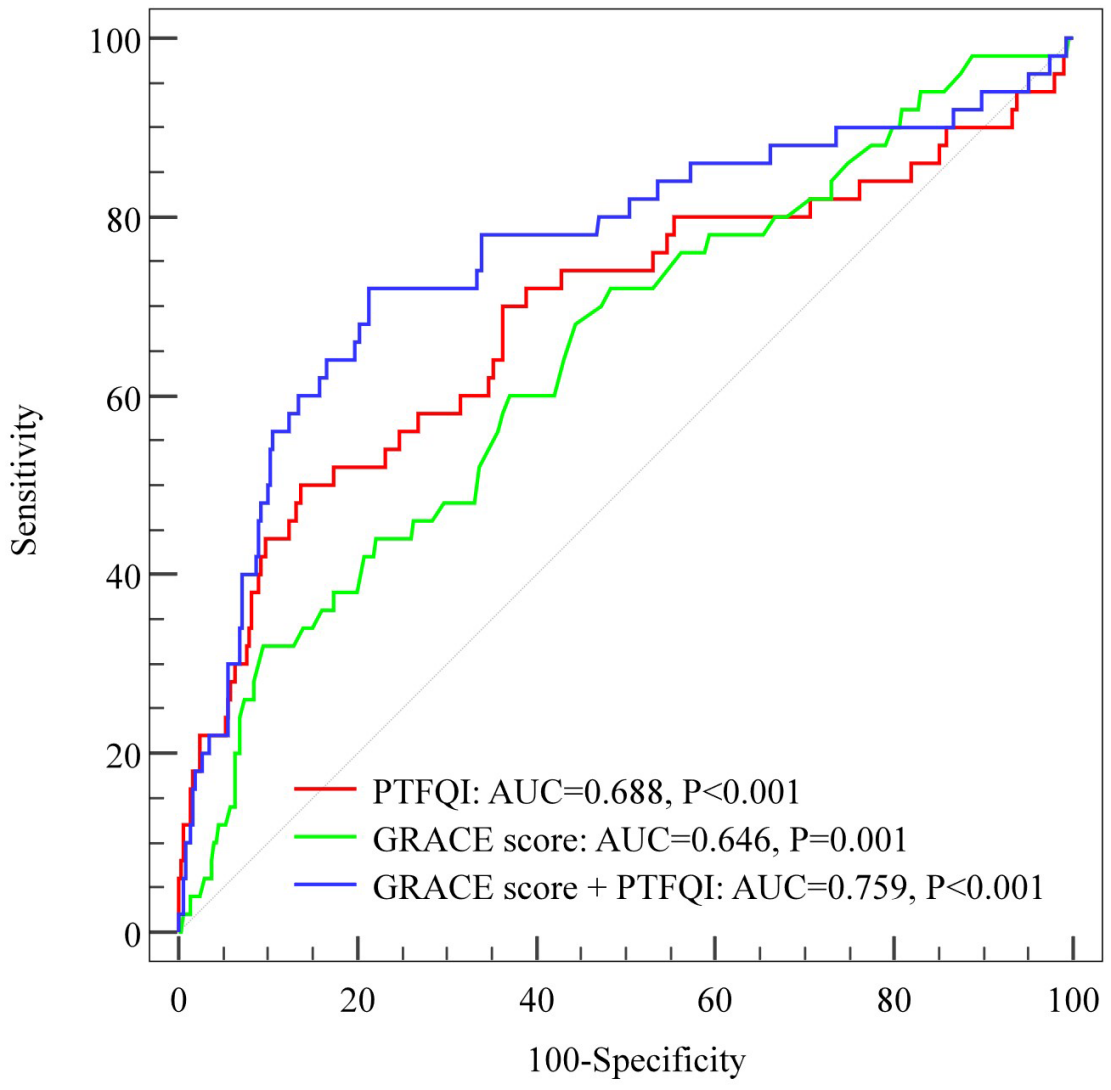
ROC curve analysis for predicting MACCEs by adding PTFQI to the GRACE risk score. The AUROC for PTFQI, GRACE score, and the combined PTFQI+GRACE score model in predicting MACCEs were 0.688 (95% CI: 0.595 - 0.780; P<0.001), 0.646 (95% CI: 0.563-0.729; P=0.001), and 0.759 (95% CI: 0.676-0.842; P<0.001), respectively. PTFQI, parametric thyroid feedback quantile-based index; GRACE score, the Global Registry of Acute Coronary Events risk score; MACCEs, major adverse cardiac and cerebrovascular events.

Adding PTFQI to the GRACE score significantly improved predictive performance for the primary endpoint compared to using the GRACE score alone, as indicated by the higher AUROC of 0.759 (95% CI 0.676-0.842) vs 0.646 (95% CI 0.563-0.729) and a statistically significant P value of 0.0108. The Z statistic of 2.549 further supported this improvement (**Figure 4**). These findings imply that PTFQI enhances the predictive capability of the GRACE score for MACCEs in patients with ACS.

### The predictive value of PTFQI for the primary and secondary endpoints

During the follow-up period, there were 17 cases of all-cause mortality (including 9 cases of cardiac death), 8 cases of stroke, 6 cases of recurrent myocardial infarction, and 27 cases of unplanned repeat revascularization. As shown in **Table 4**, in the overall study population, with respect to the cumulative incidence of secondary endpoint events, the Cox regression analysis indicated that the PTFQI was significantly associated with the prediction of nonfatal myocardial infarction (P=0.025) and unplanned repeat revascularization (P < 0.001).

**Table 4:** COX regression analysis for the association of PTFQI and risk of primary and secondary outcomes.

Events	No. of patients,n(%)	HR	95%CI	P value
MACCEs	50 (11.60)	1.290	1.158-1.437	**<0.001**
All-cause death	17 (3.94)	1.184	0.996-1.047	0.055
Cardiac death	9 (2.09)	1.144	0.904-1.449	0.262
Stroke	8 (1.86)	1.276	0.974-1.672	0.077
Myocardial infarction	6 (1.39)	1.143	0.913-1.432	**0.025**
Unplanned revascularization	27 (6.26)	1.387	1.184-1.624	**<0.001**

Bold indicates P value < 0.05. PTFQI, and parametric thyroid feedback quantile-based index; MACCEs, major adverse cardiac and cerebrovascular events.

### The predictive value of PTFQI for MACCEs in various subgroups

**Figure 5:**
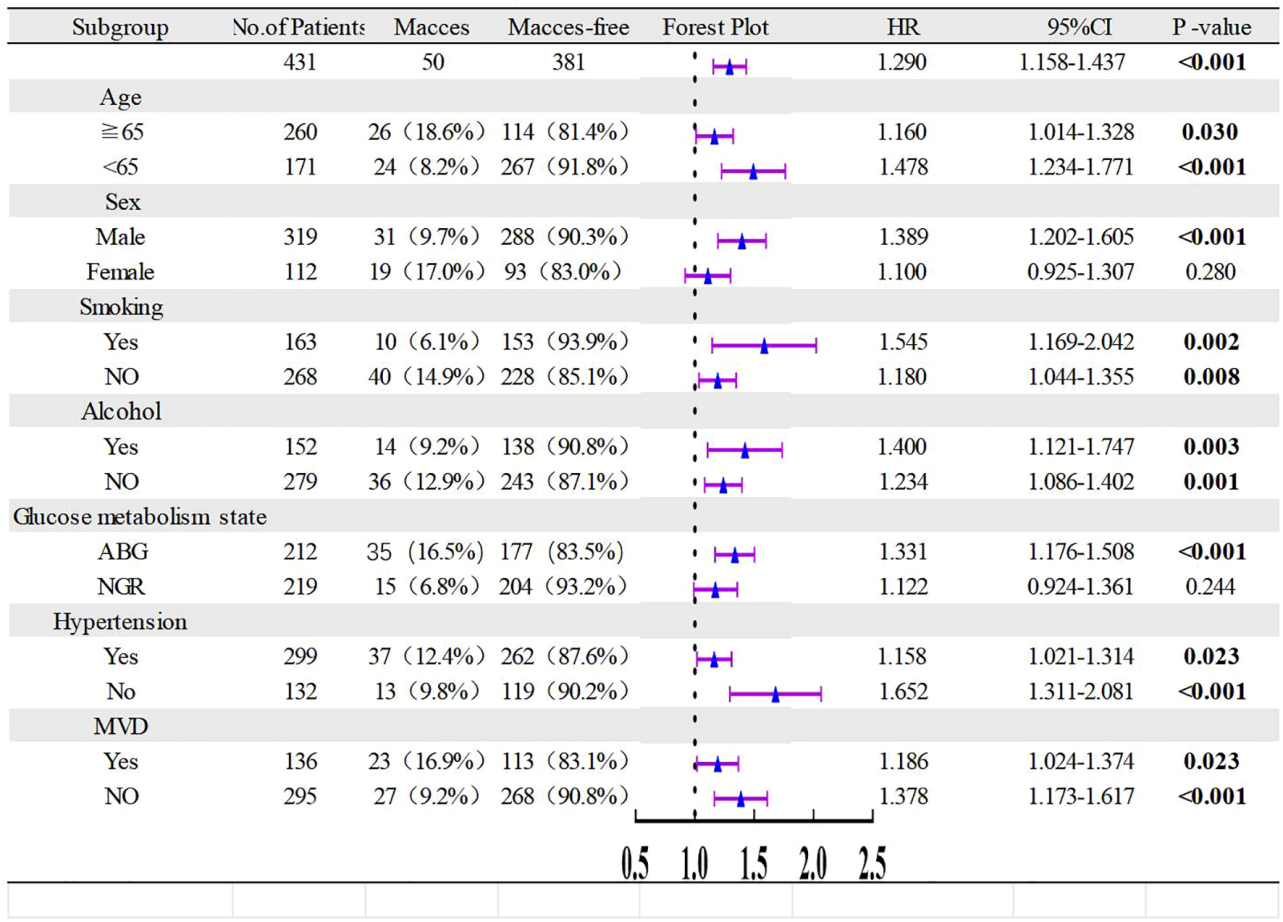
Subgroups analyses of PTFQI for MACCEs. HR, hazard ratio; CI, confidence interval; ABG, Abnormal blood glucose; NGR, normal glucose regulation; MVD, multivessel disease.

Subgroup analyses were conducted to evaluate the predictive capacity of the PTFQI across various demographic characteristics and comorbidities. The results indicate that the PTFQI was more predictive in males and patients with abnormal blood glucose, while it has limited predictive value in females and patients with normal blood glucose. Notably, it remained a significant predictor of MACCEs regardless of age ≥65 years, current smoking, hypertension, or MVD (**Figure 5**).

## Discussion

In this cohort study conducted, we identified a positive correlation between MACCEs and impaired thyroid hormone sensitivity (elevated TSHI, TT4RI, TFQI, and PTFQI) in ACS patients undergoing PCI. Even after adjusting for potential confounding factors, thyroid hormone sensitivity indices remained significant predictors of MACCEs. Adding PTFQI to the GRACE score significantly improved the predictive performance for the primary endpoint. Notable associations between PTFQI and MACCEs were observed in males, individuals with abnormal blood glucose.

The hypothesis that thyroid indicators can predict MACCEs has been strongly supported, which is mediated through thyroid-induced cardiac oxidative stress, inflammatory response, and remodeling-related pathophysiological mechanisms. Excessive THs lead to increased production of reactive oxygen species surpassing the antioxidant defense capabilities (such as superoxide dismutase, glutathione peroxidase), resulting in oxidative damage. Conversely, in hypothyroidism, the activity of antioxidants is decreased, indirectly promoting oxidative stress (16). Excessive thyroid hormones lead to increased production. Oxidative stress damages vascular endothelial cells, reduces the bioavailability of nitric oxide promotes LDL oxidation, endothelial damage, accelerates the formation of atherosclerotic plaques, potentially leading to myocardial infarction or decompensated heart failure (9, 17). Furthermore, THs disturbances activate the release of inflammatory factors (such as IL-1, IL-2, IL-6, and TNF-α) through oxidative stress (18, 19), and chronic inflammation is a core mechanism of atherosclerosis, promoting plaque instability and thrombus formation, exacerbating myocardial remodeling and ischemia-reperfusion injury. The imbalance of thyroid hormones, accumulation of reactive oxygen species and inflammation collectively lead to myocardial hypertrophy, fibrosis, and impaired contractile function (17). THs induce cardiac remodeling through multiple pathways, at the genomic level, THs regulate gene transcription through nuclear receptors (TRα and TRβ), with overexpression inducing cardiomyocyte hypertrophy leading to chamber dilation and subsequent heart failure (20, 21). THs impact cardiac contractile function through regulating the transcription of contractile proteins (such as upregulation of α-myosin heavy chain and downregulation of β-myosin heavy chain) and ion channels (such as SERCA2, Na+/K+-ATPase) (22, 23). Non-genomically, THs activate the PI3K/Akt/mTOR pathway, promoting physiological hypertrophy (23). Studies have shown that THs reverse molecular remodeling changes by restoring the expression of MHC, SERCA, BNP, among others, leading to improvements in coronary blood flow, prevention of pathological progression, improvement in functional status, and prevention of left ventricular dysfunction/remodeling (24, 25). However, excess THs can increase the risk of arrhythmias (such as atrial fibrillation) through the inward rectifier K+ currents, leading to increased risk of cardiovascular and cerebral thrombotic events. THs stimulate mitochondrial biogenesis through various mechanisms, promoting ATP production, increasing aerobic metabolism, and generating cardiac protective effects through glucose oxidation (26), but excess THs can cause mitochondrial damage and energy consumption, leading to cardiac dysfunction (27). Overall, in appropriate amounts, THs can enhance cardiac function, but in cases of homeostatic imbalance, they can lead to pathological changes through multiple mechanisms, resulting in MACCEs.

Recent studies have indicated that thyroid hormone resistance in the euthyroid population is linked to BMI, blood pressure, uric acid levels, blood lipids, diabetes, metabolic syndrome, the TyG index, Hcy, and an elevated risk of carotid plaque (13, 14, 28, 29). These findings suggest that an upregulated thyroid axis is intricately associated with the development and progression of CAD. A study has demonstrated a positive correlation between stroke risk in patients with CAD and TSHI, TFQI, and PTFQI, revealing higher odds ratios among individuals under 65 years of age, males, and those with diabetes (30). In a prospective cohort study suggested that TT4RI, TSHI, and TFQI were identified as markers of decreased sensitivity to THs, correlating with elevated risks of major adverse cardiac events and cardiovascular mortality in euthyroid individuals undergoing coronary angiography (31).

Our study corroborated the findings of the above studies. However, it deviated in terms of patient cohorts, as our study focused on individuals treated with PCI, and it was validated by the PTFQI. The AUROC indicates a slight superiority of PTFQI at 0.688 over TSHI at 0.676 and TT4RI at 0.674. It is worth noting that TSHI and TT4RI are calculated based on measurements of FT4 and TSH, while TFQI and PTFQI rank FT4 and TSH from minimum to maximum and convert them into percentiles between 0 and 1 using a cumulative distribution function with population data on hormone levels. Compared to TT4RI and TSHI, TFQI and PTFQI exhibit stability in cases of thyroid-related primary clinical hyperthyroidism or hypothyroidism, without extreme values. Moreover, PTFQI demonstrates versatility across diverse populations and increased clinical relevance.

The results of this study demonstrate a close association between changes in thyroid hormone sensitivity and adverse outcomes in patients with ACS, and the underlying molecular mechanisms remain unclear. This relationship may be due to (1) the different distributions of THs receptors (beta-receptors predominant in thyrotrophic cells and alpha-receptors predominant in myocytes) (32). However, FT4 levels may not be sufficient to suppress TSH secretion at the pituitary gland, and they are adequate to potentially induce harmful effects on cardiac function. (2) MI may, in turn, affect THs homeostasis by regulating the expression of thyroid-related genes in the brain, and ischemia and hypoxia-induced damage to the pituitary gland can increase thyroid hormone resistance. Yang et al. (33) reported that decreased sensitivity to THs, represented by an increased level of PTFQI, is linked with impaired renal function, further leading to water and sodium retention. Moreover, emerging evidence indicates that patients with elevated PTFQI levels are significantly associated with an increased incidence of type 2 diabetes, ischemic heart disease, atrial fibrillation, and hypertension (34, 35). The above findings help elucidate our results, however, the specific molecular mechanisms await further confirmation and clarification.

We found that the thyroid hormone resistance index significantly increased with age. Although all analyses were adjusted for age and other potential confounding factors, some residual confounders may still exist. Given that many factors may be related to CAD, it is not possible to adjust for unavailable variables and unknown factors; therefore, potential residual confounding factors must be considered when interpreting the study results. Subgroup analysis revealed that thyroid hormone resistance was pre-significant for MACCEs in male patients, which may be related to the differential feedback regulation influenced by the HPT axis hormones. Testosterone may impact repolarization by regulating ion channels, while estrogen may have anti-fibrotic effects, preventing apoptosis and necrosis in heart and endothelial cells (36, 37). However, this study did not measure sex hormone levels, warranting further investigation for verification. In patients with abnormal blood glucose, there is a significant predictive value, while the prediction is less significant in normoglycemic patients. This could be attributed to the increase in antithyroid resistance markers with aging, similar to diabetes, and the medication status of diabetic patients can also affect thyroid hormone sensitivity (38). Given the potential relationship of numerous factors with diabetes and metabolic syndrome, adjusting for unavailable variables and unknown factors becomes unfeasible. Consequently, one must take into account the potential residual confounding when interpreting the study findings. CAD requires comprehensive clinical management, involving not only the control of blood glucose, blood pressure, and blood lipids but also the focus on energy balance. Monitoring resistance to THs could be utilized to assess the intentional reversal of abnormal energy balance.

Previous research has demonstrated that models combining anatomical and clinical factors can enhance discriminative capacity and offer improved risk assessment. A retrospective analysis further showed that unsupervised learning can identify four clinical phenotypes in patients with normal myocardial perfusion imaging, among whom a subset remains at high risk for MI or death. This underscores the utility of patient phenotyping in risk stratification for individuals with normal imaging results (39). This study demonstrates that PTFQI can enhance the predictive value of GRACE score for MACCEs. However, current literature on the diagnosis and treatment of CAD lacks the inclusion of thyroid hormone resistance markers in established risk prediction tools such as the GRACE score. Consequently, we recommend incorporating thyroid hormone sensitivity indicators, unsupervised learning, or other methods in future research to develop or update existing risk prediction models, potentially enhancing clinicians’ ability to differentiate high-risk CAD patients.

### Limitations

This study has certain limitations. First, due to limitations under China’s medical insurance policy, most patients did not undergo TSH and FT4 testing, making it impossible to calculate relevant indices. As a result, a large number of cases were excluded, upon comparing included and excluded cases, no notable disparities were found in terms of demographic characteristics and comorbidities (**Supplementary Table 5**). Moreover, variations in thyroid function among populations in different regions further constrained the generalizability of the research findings. Second, although we controlled for essential demographic and behavioral factors, in the baseline table, we included LDL-C and HDL-C, but there was no statistical difference between the two groups. Furthermore, since C-reactive protein and IL-6, as inflammatory markers, were not routinely tested in clinical practice and thus were not included in the multivariate analysis, this omission may lead to an overestimation or underestimation of the true association between impaired thyroid hormone sensitivity and major adverse cardiovascular events. We cannot rule out other conditions, such as immune disorders, which could also explain the coincidence of elevated THs due to autoimmune thyroid diseases and latent autoimmune diabetes in adults. However, from a population perspective, energy imbalance is more prevalent. Third, the study overlooked peripheral sensitivity to THs and the influence of pituitary deiodinases, which play crucial roles in converting THs into active and inactive forms, as integral components of thyroid synthesis and regulation. And thyroid function was subsequently measured only once before PCI, and dynamic monitoring was lacking. Notably, the incidence of ACS increases in association with COVID-19, which is attributed to coronary artery thrombosis and myocardial oxygen deprivation. The co-occurrence of COVID-19 and ACS, compounded by underlying comorbidities, poses challenges in diagnosing ACS within the context of COVID-19. The prevalence of complications following AMI is high, leading to increased mortality rates (40). Therefore, the results must be interpreted carefully because this sample does not fully represent the general population.

In the future, higher-quality clinical and basic research is needed to elucidate the impact of thyroid hormone sensitivity on CAD; combining inflammatory markers, endothelial function, and thyroid hormone sensitivity indices to predict CAD risk; exploring interventions targeting thyroid hormone sensitivity, as well as the role of thyroid hormone analogs or thyroid hormone receptor agonists in improving cardiovascular function, may become a completely new strategy in the prevention and treatment of CAD.

## Conclusions

The thyroid hormone sensitivity indices, serving as a marker of metabolic irregularities, demonstrates a significant positive correlation with the risk of MACCEs following PCI in patients with ACS. Conducting routine thyroid hormone evaluations before PCI and assessing thyroid hormone sensitivity indices can aid in early identification of high-risk individuals and the customization of preventative and treatment strategies.

## Data Availability

The raw data supporting the conclusions of this article will be made available by the authors, without undue reservation.
