# Supplementary material for: Predicting major cardiac and cerebrovascular events in acute coronary syndrome patients using the thyroid hormone sensitivity index
Source: Front Endocrinol (Lausanne). 2025 Jul 2;16:1543378. doi: 10.3389/fendo.2025.1543378 (PMC12264728; doi:10.3389/fendo.2025.1543378)
Supplement: Supplementary file 1 [file Table1.docx]

**Table 1 Univariate and multivariate Cox regression analysis for TSHI predicting the primary endpoint**

|  | **Univariate Analysis** | | |  | **Multivariate Analysis** | | |
| --- | --- | --- | --- | --- | --- | --- | --- |
|  | **HR** | **95% CI** | **P value** |  | **HR** | **95% CI** | **P value** |
| Male | 0.439 | 0.245-0.788 | **0.006** |  | 1.388 | 0.670-2.875 | 0.377 |
| Age | 1.984 | 1.120-3.517 | **0.019** |  | 1.010 | 0.970-1.052 | 0.617 |
| BMI | 1.024 | 0.942-1.114 | 0.572 |  |  |  |  |
| Smoking | 0.300 | 0.145-0.619 | **0.001** |  | 0.555 | 0.224-1.373 | 0.203 |
| Alcohol | 0.641 | 0.339-1.210 | 0.170 |  |  |  |  |
| Diabetes mellitus | 1.988 | 1.126-3.058 | **0.018** |  | 1.802 | 0.920-3.530 | 0.086 |
| Hypertension | 1.640 | 0.848-3.171 | 0.141 |  |  |  |  |
| Previous CHD | 1.582 | 0.834-3.003 | 0.160 |  |  |  |  |
| Previous angina pectoris | 0.951 | 0.495-1.830 | 0.881 |  |  |  |  |
| Previous MI | 0.467 | 0.064-3.397 | 0.452 |  |  |  |  |
| Previous PCI | 1.176 | 0.422-3.279 | 0.756 |  |  |  |  |
| Previous COPD | 1.926 | 0.463-8.014 | 0.368 |  |  |  |  |
| Atrial fibrillation | 0.048 | 0.000-870.298 | 0.544 |  |  |  |  |
| Stroke | 1.164 | 0.357-3.789 | 0.801 |  |  |  |  |
| Chronic kidney diseases | 0.048 | 0.000-1844.463 | 0.574 |  |  |  |  |
| UA | 1.178 | 0.670-2.072 | 0.569 |  |  |  |  |
| STEMI | 0.476 | 0.214-1.062 | 0.070 |  |  |  |  |
| NSTEMI | 1.483 | 0.795-2.769 | 0.216 |  |  |  |  |
| SBP | 1.002 | 0.990-1.016 | 0.709 |  |  |  |  |
| DBP | 0.986 | 0.966-1.007 | 0.192 |  |  |  |  |
| Heart rate | 0.995 | 0.975-1.016 | 0.660 |  |  |  |  |
| Cardiogenic shock | 2.346 | 0.566-9.714 | 0.298 |  |  |  |  |
| BNP | 1.001 | 1.000-1.001 | **0.003** |  | 1.000 | 1.000-1.001 | 0.266 |
| Creatinine clearance rate | 0.986 | 0.975-0.997 | **0.012** |  | 0.996 | 0.979-1.012 | 0.608 |
| Fasting blood-glucose | 1.060 | 0.976-1.152 | 0.168 |  |  |  |  |
| Triglyceride | 0.905 | 0.716-1.144 | 0.406 |  |  |  |  |
| Total cholesterol | 0.991 | 0.905-1.086 | 0.852 |  |  |  |  |
| LDL-C | 1.008 | 0.732-1.387 | 0.962 |  |  |  |  |
| HDL-C | 0.914 | 0.560-1.493 | 0.720 |  |  |  |  |
| apoA | 0.893 | 0.392-2.031 | 0.787 |  |  |  |  |
| apoB | 1.071 | 0.380-3.018 | 0.897 |  |  |  |  |
| Hcy | 1.005 | 0.995-1.015 | 0.340 |  |  |  |  |
| Lp（a） | 1.000 | 0.999-1.001 | 0.900 |  |  |  |  |
| Albumin | 0.969 | 0.905-1.037 | 0.358 |  |  |  |  |
| Fib | 1.252 | 1.113-1.408 | **<0.001** |  | 1.258 | 1.094-1.448 | **0.001** |
| D-D | 1.008 | 0.961-1.057 | 0.741 |  |  |  |  |
| PT | 0.993 | 0.941-1.048 | 0.797 |  |  |  |  |
| INR | 0.928 | 0.352-2.448 | 0.880 |  |  |  |  |
| APTT | 0.997 | 0.989-1.005 | 0.471 |  |  |  |  |
| Hemoglobin | 1.004 | 1.000-1.008 | 0.076 |  |  |  |  |
| PLT count | 0.998 | 0.993-1.003 | 0.416 |  |  |  |  |
| WBC count | 0.921 | 0.805-1.055 | 0.236 |  |  |  |  |
| T3 | 0.821 | 0.257-2.619 | 0.739 |  |  |  |  |
| T4 | 0.994 | 0.978-1.009 | 0.433 |  |  |  |  |
| TSH | 1.017 | 1.008-1.026 | **<0.001** |  |  |  |  |
| FT3 | 1.019 | 0.857-1.212 | 0.828 |  |  |  |  |
| FT4 | 1.033 | 1.010-1.057 | **0.005** |  |  |  |  |
| FT3/FT4 | 1.161 | 0.665-2.026 | 0.600 |  |  |  |  |
| TSHI | 1.331 | 1.183-1.498 | **<0.001** |  | 1.277 | 1.110-1.468 | **<0.001** |
| TT4RI | 1.001 | 1.001-1.002 | **<0.001** |  |  |  |  |
| TFQI | 1.173 | 1.087-1.266 | **<0.001** |  |  |  |  |
| PTFQI | 1.290 | 1.158-1.437 | **<0.001** |  |  |  |  |
| LVEF | 0.988 | 0.957-1.020 | 0.452 |  |  |  |  |
| MVD | 2.014 | 1.141-3.556 | **0.016** |  | 1.274 | 0.662-2.454 | 0.468 |
| LM | 0.680 | 0.202-2.293 | 0.534 |  |  |  |  |
| Branches lesions | 1.388 | 0.467-4.125 | 0.555 |  |  |  |  |
| Calcified lesions | 1.446 | 0.673-3.108 | 0.344 |  |  |  |  |
| Thrombosis | 0.424 | 0.098-1.841 | 0.252 |  |  |  |  |
| CTO | 0.919 | 0.397-2.125 | 0.843 |  |  |  |  |
| Long lesion | 1.652 | 0.567-4.819 | 0.358 |  |  |  |  |
| Coronary rotablation | 1.817 | 0.541-6.105 | 0.334 |  |  |  |  |
| Thrombus aspiration | 0.047 | 0.000-84.191 | 0.428 |  |  |  |  |
| IVUS | 1.603 | 0.497-5.174 | 0.430 |  |  |  |  |
| Aspirin | 0.527 | 0.127-2.190 | 0.378 |  |  |  |  |
| Clopidogrel | 0.652 | 0.089-4.777 | 0.674 |  |  |  |  |
| Anticoagulation | 2.092 | 0.503-8.704 | 0.310 |  |  |  |  |
| Statins | 0.526 | 0.127-2.177 | 0.375 |  |  |  |  |
| Diuretics | 2.219 | 1.172-4.202 | **0.014** |  | 1.409 | 0.585-3.393 | 0.444 |
| β-blocker | 0.928 | 0.514-1.673 | 0.803 |  |  |  |  |
| ACEI/ARB | 1.690 | 0.953-3.000 | 0.073 |  |  |  |  |
| Insulin treatment | 2.564 | 1.236-5.231 | **0.011** |  | 1.245 | 0.502-3.087 | 0.637 |

**Table 2 Univariate and multivariate Cox regression analysis for TT4RI predicting the primary endpoint**

|  | **Univariate Analysis** | | |  | **Multivariate Analysis** | | |
| --- | --- | --- | --- | --- | --- | --- | --- |
|  | **HR** | **95% CI** | **P value** |  | **HR** | **95% CI** | **P value** |
| Male | 0.439 | 0.245-0.788 | **0.006** |  | 1.596 | 0.778-3.276 | 0.202 |
| Age | 1.984 | 1.120-3.517 | **0.019** |  | 1.006 | 0.966-1.048 | 0.769 |
| BMI | 1.024 | 0.942-1.114 | 0.572 |  |  |  |  |
| Smoking | 0.300 | 0.145-0.619 | **0.001** |  | 0.467 | 0.185-1.180 | 0.203 |
| Alcohol | 0.641 | 0.339-1.210 | 0.170 |  |  |  |  |
| Diabetes mellitus | 1.988 | 1.126-3.058 | **0.018** |  | 1.802 | 0.920-3.530 | 0.107 |
| Hypertension | 1.640 | 0.848-3.171 | 0.141 |  |  |  |  |
| Previous CHD | 1.582 | 0.834-3.003 | 0.160 |  |  |  |  |
| Previous angina pectoris | 0.951 | 0.495-1.830 | 0.881 |  |  |  |  |
| Previous MI | 0.467 | 0.064-3.397 | 0.452 |  |  |  |  |
| Previous PCI | 1.176 | 0.422-3.279 | 0.756 |  |  |  |  |
| Previous COPD | 1.926 | 0.463-8.014 | 0.368 |  |  |  |  |
| Atrial fibrillation | 0.048 | 0.000-870.298 | 0.544 |  |  |  |  |
| Stroke | 1.164 | 0.357-3.789 | 0.801 |  |  |  |  |
| Chronic kidney diseases | 0.048 | 0.000-1844.463 | 0.574 |  |  |  |  |
| UA | 1.178 | 0.670-2.072 | 0.569 |  |  |  |  |
| STEMI | 0.476 | 0.214-1.062 | 0.070 |  |  |  |  |
| NSTEMI | 1.483 | 0.795-2.769 | 0.216 |  |  |  |  |
| SBP | 1.002 | 0.990-1.016 | 0.709 |  |  |  |  |
| DBP | 0.986 | 0.966-1.007 | 0.192 |  |  |  |  |
| Heart rate | 0.995 | 0.975-1.016 | 0.660 |  |  |  |  |
| Cardiogenic shock | 2.346 | 0.566-9.714 | 0.298 |  |  |  |  |
| BNP | 1.001 | 1.000-1.001 | **0.003** |  | 1.000 | 1.000-1.001 | 0.269 |
| Creatinine clearance rate | 0.986 | 0.975-0.997 | **0.012** |  | 0.996 | 0.980-1.012 | 0.619 |
| Fasting blood-glucose | 1.060 | 0.976-1.152 | 0.168 |  |  |  |  |
| Triglyceride | 0.905 | 0.716-1.144 | 0.406 |  |  |  |  |
| Total cholesterol | 0.991 | 0.905-1.086 | 0.852 |  |  |  |  |
| LDL-C | 1.008 | 0.732-1.387 | 0.962 |  |  |  |  |
| HDL-C | 0.914 | 0.560-1.493 | 0.720 |  |  |  |  |
| apoA | 0.893 | 0.392-2.031 | 0.787 |  |  |  |  |
| apoB | 1.071 | 0.380-3.018 | 0.897 |  |  |  |  |
| Hcy | 1.005 | 0.995-1.015 | 0.340 |  |  |  |  |
| Lp（a） | 1.000 | 0.999-1.001 | 0.900 |  |  |  |  |
| Albumin | 0.969 | 0.905-1.037 | 0.358 |  |  |  |  |
| Fib | 1.252 | 1.113-1.408 | **<0.001** |  | 1.288 | 1.125-1.476 | **<0.001** |
| D-D | 1.008 | 0.961-1.057 | 0.741 |  |  |  |  |
| PT | 0.993 | 0.941-1.048 | 0.797 |  |  |  |  |
| INR | 0.928 | 0.352-2.448 | 0.880 |  |  |  |  |
| APTT | 0.997 | 0.989-1.005 | 0.471 |  |  |  |  |
| Hemoglobin | 1.004 | 1.000-1.008 | 0.076 |  |  |  |  |
| PLT count | 0.998 | 0.993-1.003 | 0.416 |  |  |  |  |
| WBC count | 0.921 | 0.805-1.055 | 0.236 |  |  |  |  |
| T3 | 0.821 | 0.257-2.619 | 0.739 |  |  |  |  |
| T4 | 0.994 | 0.978-1.009 | 0.433 |  |  |  |  |
| TSH | 1.017 | 1.008-1.026 | **<0.001** |  |  |  |  |
| FT3 | 1.019 | 0.857-1.212 | 0.828 |  |  |  |  |
| FT4 | 1.033 | 1.010-1.057 | **0.005** |  |  |  |  |
| FT3/FT4 | 1.161 | 0.665-2.026 | 0.600 |  |  |  |  |
| TSHI | 1.331 | 1.183-1.498 | **<0.001** |  |  |  |  |
| TT4RI | 1.001 | 1.001-1.002 | **<0.001** |  | 1.002 | 1.001-1.003 | **<0.001** |
| TFQI | 1.173 | 1.087-1.266 | **<0.001** |  |  |  |  |
| PTFQI | 1.290 | 1.158-1.437 | **<0.001** |  |  |  |  |
| LVEF | 0.988 | 0.957-1.020 | 0.452 |  |  |  |  |
| MVD | 2.014 | 1.141-3.556 | **0.016** |  | 1.344 | 0.695-2.598 | 0.379 |
| LM | 0.680 | 0.202-2.293 | 0.534 |  |  |  |  |
| Branches lesions | 1.388 | 0.467-4.125 | 0.555 |  |  |  |  |
| Calcified lesions | 1.446 | 0.673-3.108 | 0.344 |  |  |  |  |
| Thrombosis | 0.424 | 0.098-1.841 | 0.252 |  |  |  |  |
| CTO | 0.919 | 0.397-2.125 | 0.843 |  |  |  |  |
| Long lesion | 1.652 | 0.567-4.819 | 0.358 |  |  |  |  |
| Coronary rotablation | 1.817 | 0.541-6.105 | 0.334 |  |  |  |  |
| Thrombus aspiration | 0.047 | 0.000-84.191 | 0.428 |  |  |  |  |
| IVUS | 1.603 | 0.497-5.174 | 0.430 |  |  |  |  |
| Aspirin | 0.527 | 0.127-2.190 | 0.378 |  |  |  |  |
| Clopidogrel | 0.652 | 0.089-4.777 | 0.674 |  |  |  |  |
| Anticoagulation | 2.092 | 0.503-8.704 | 0.310 |  |  |  |  |
| Statins | 0.526 | 0.127-2.177 | 0.375 |  |  |  |  |
| Diuretics | 2.219 | 1.172-4.202 | **0.014** |  | 1.491 | 0.620-3.588 | 0.372 |
| β-blocker | 0.928 | 0.514-1.673 | 0.803 |  |  |  |  |
| ACEI/ARB | 1.690 | 0.953-3.000 | 0.073 |  |  |  |  |
| Insulin treatment | 2.564 | 1.236-5.231 | **0.011** |  | 1.149 | 0.459-2.874 | 0.767 |

**Table 3 Univariate and multivariate Cox regression analysis for TFQI predicting the primary endpoint**

|  | **Univariate Analysis** | | |  | **Multivariate Analysis** | | |
| --- | --- | --- | --- | --- | --- | --- | --- |
|  | **HR** | **95% CI** | **P value** |  | **HR** | **95% CI** | **P value** |
| Male | 0.439 | 0.245-0.788 | **0.006** |  | 1.442 | 0.694-2.999 | 0.327 |
| Age | 1.984 | 1.120-3.517 | **0.019** |  | 1.004 | 0.963-1.046 | 0.863 |
| BMI | 1.024 | 0.942-1.114 | 0.572 |  |  |  |  |
| Smoking | 0.300 | 0.145-0.619 | **0.001** |  | 0.641 | 0.258-1.593 | 0.338 |
| Alcohol | 0.641 | 0.339-1.210 | 0.170 |  |  |  |  |
| Diabetes mellitus | 1.988 | 1.126-3.058 | **0.018** |  | 1.811 | 0.922-3.555 | 0.084 |
| Hypertension | 1.640 | 0.848-3.171 | 0.141 |  |  |  |  |
| Previous CHD | 1.582 | 0.834-3.003 | 0.160 |  |  |  |  |
| Previous angina pectoris | 0.951 | 0.495-1.830 | 0.881 |  |  |  |  |
| Previous MI | 0.467 | 0.064-3.397 | 0.452 |  |  |  |  |
| Previous PCI | 1.176 | 0.422-3.279 | 0.756 |  |  |  |  |
| Previous COPD | 1.926 | 0.463-8.014 | 0.368 |  |  |  |  |
| Atrial fibrillation | 0.048 | 0.000-870.298 | 0.544 |  |  |  |  |
| Stroke | 1.164 | 0.357-3.789 | 0.801 |  |  |  |  |
| Chronic kidney diseases | 0.048 | 0.000-1844.463 | 0.574 |  |  |  |  |
| UA | 1.178 | 0.670-2.072 | 0.569 |  |  |  |  |
| STEMI | 0.476 | 0.214-1.062 | 0.070 |  |  |  |  |
| NSTEMI | 1.483 | 0.795-2.769 | 0.216 |  |  |  |  |
| SBP | 1.002 | 0.990-1.016 | 0.709 |  |  |  |  |
| DBP | 0.986 | 0.966-1.007 | 0.192 |  |  |  |  |
| Heart rate | 0.995 | 0.975-1.016 | 0.660 |  |  |  |  |
| Cardiogenic shock | 2.346 | 0.566-9.714 | 0.298 |  |  |  |  |
| BNP | 1.001 | 1.000-1.001 | **0.003** |  | 1.000 | 1.000-1.001 | 0.430 |
| Creatinine clearance rate | 0.986 | 0.975-0.997 | **0.012** |  | 0.995 | 0.978-1.012 | 0.531 |
| Fasting blood-glucose | 1.060 | 0.976-1.152 | 0.168 |  |  |  |  |
| Triglyceride | 0.905 | 0.716-1.144 | 0.406 |  |  |  |  |
| Total cholesterol | 0.991 | 0.905-1.086 | 0.852 |  |  |  |  |
| LDL-C | 1.008 | 0.732-1.387 | 0.962 |  |  |  |  |
| HDL-C | 0.914 | 0.560-1.493 | 0.720 |  |  |  |  |
| apoA | 0.893 | 0.392-2.031 | 0.787 |  |  |  |  |
| apoB | 1.071 | 0.380-3.018 | 0.897 |  |  |  |  |
| Hcy | 1.005 | 0.995-1.015 | 0.340 |  |  |  |  |
| Lp（a） | 1.000 | 0.999-1.001 | 0.900 |  |  |  |  |
| Albumin | 0.969 | 0.905-1.037 | 0.358 |  |  |  |  |
| Fib | 1.252 | 1.113-1.408 | **<0.001** |  | 1.311 | 1.136-1.512 | **<0.001** |
| D-D | 1.008 | 0.961-1.057 | 0.741 |  |  |  |  |
| PT | 0.993 | 0.941-1.048 | 0.797 |  |  |  |  |
| INR | 0.928 | 0.352-2.448 | 0.880 |  |  |  |  |
| APTT | 0.997 | 0.989-1.005 | 0.471 |  |  |  |  |
| Hemoglobin | 1.004 | 1.000-1.008 | 0.076 |  |  |  |  |
| PLT count | 0.998 | 0.993-1.003 | 0.416 |  |  |  |  |
| WBC count | 0.921 | 0.805-1.055 | 0.236 |  |  |  |  |
| T3 | 0.821 | 0.257-2.619 | 0.739 |  |  |  |  |
| T4 | 0.994 | 0.978-1.009 | 0.433 |  |  |  |  |
| TSH | 1.017 | 1.008-1.026 | **<0.001** |  |  |  |  |
| FT3 | 1.019 | 0.857-1.212 | 0.828 |  |  |  |  |
| FT4 | 1.033 | 1.010-1.057 | **0.005** |  |  |  |  |
| FT3/FT4 | 1.161 | 0.665-2.026 | 0.600 |  |  |  |  |
| TSHI | 1.331 | 1.183-1.498 | **<0.001** |  |  |  |  |
| TT4RI | 1.001 | 1.001-1.002 | **<0.001** |  |  |  |  |
| TFQI | 1.173 | 1.087-1.266 | **<0.001** |  | 1.130 | 1.043-1.224 | **0.003** |
| PTFQI | 1.290 | 1.158-1.437 | **<0.001** |  |  |  |  |
| LVEF | 0.988 | 0.957-1.020 | 0.452 |  |  |  |  |
| MVD | 2.014 | 1.141-3.556 | **0.016** |  | 1.386 | 0.718-2.675 | 0.331 |
| LM | 0.680 | 0.202-2.293 | 0.534 |  |  |  |  |
| Branches lesions | 1.388 | 0.467-4.125 | 0.555 |  |  |  |  |
| Calcified lesions | 1.446 | 0.673-3.108 | 0.344 |  |  |  |  |
| Thrombosis | 0.424 | 0.098-1.841 | 0.252 |  |  |  |  |
| CTO | 0.919 | 0.397-2.125 | 0.843 |  |  |  |  |
| Long lesion | 1.652 | 0.567-4.819 | 0.358 |  |  |  |  |
| Coronary rotablation | 1.817 | 0.541-6.105 | 0.334 |  |  |  |  |
| Thrombus aspiration | 0.047 | 0.000-84.191 | 0.428 |  |  |  |  |
| IVUS | 1.603 | 0.497-5.174 | 0.430 |  |  |  |  |
| Aspirin | 0.527 | 0.127-2.190 | 0.378 |  |  |  |  |
| Clopidogrel | 0.652 | 0.089-4.777 | 0.674 |  |  |  |  |
| Anticoagulation | 2.092 | 0.503-8.704 | 0.310 |  |  |  |  |
| Statins | 0.526 | 0.127-2.177 | 0.375 |  |  |  |  |
| Diuretics | 2.219 | 1.172-4.202 | **0.014** |  | 1.221 | 0.511-2.920 | 0.653 |
| β-blocker | 0.928 | 0.514-1.673 | 0.803 |  |  |  |  |
| ACEI/ARB | 1.690 | 0.953-3.000 | 0.073 |  |  |  |  |
| Insulin treatment | 2.564 | 1.236-5.231 | **0.011** |  | 1.214 | 0.492-2.992 | 0.674 |

**Table 4 Univariate and multivariate Cox regression analysis for PTFQI predicting the primary endpoint**

|  | **Univariate Analysis** | | |  | **Multivariate Analysis** | | |
| --- | --- | --- | --- | --- | --- | --- | --- |
|  | **HR** | **95% CI** | **P value** |  | **HR** | **95% CI** | **P value** |
| Male | 0.439 | 0.245-0.788 | **0.006** |  | 1.299 | 0.624-2.704 | 0.484 |
| Age | 1.984 | 1.120-3.517 | **0.019** |  | 1.004 | 0.964-1.047 | 0.835 |
| BMI | 1.024 | 0.942-1.114 | 0.572 |  |  |  |  |
| Smoking | 0.300 | 0.145-0.619 | **0.001** |  | 0.697 | 0.276-1.762 | 0.446 |
| Alcohol | 0.641 | 0.339-1.210 | 0.170 |  |  |  |  |
| Diabetes mellitus | 1.988 | 1.126-3.058 | **0.018** |  | 1.889 | 0.967-3.693 | 0.063 |
| Hypertension | 1.640 | 0.848-3.171 | 0.141 |  |  |  |  |
| Previous CHD | 1.582 | 0.834-3.003 | 0.160 |  |  |  |  |
| Previous angina pectoris | 0.951 | 0.495-1.830 | 0.881 |  |  |  |  |
| Previous MI | 0.467 | 0.064-3.397 | 0.452 |  |  |  |  |
| Previous PCI | 1.176 | 0.422-3.279 | 0.756 |  |  |  |  |
| Previous COPD | 1.926 | 0.463-8.014 | 0.368 |  |  |  |  |
| Atrial fibrillation | 0.048 | 0.000-870.298 | 0.544 |  |  |  |  |
| Stroke | 1.164 | 0.357-3.789 | 0.801 |  |  |  |  |
| Chronic kidney diseases | 0.048 | 0.000-1844.463 | 0.574 |  |  |  |  |
| UA | 1.178 | 0.670-2.072 | 0.569 |  |  |  |  |
| STEMI | 0.476 | 0.214-1.062 | 0.070 |  |  |  |  |
| NSTEMI | 1.483 | 0.795-2.769 | 0.216 |  |  |  |  |
| SBP | 1.002 | 0.990-1.016 | 0.709 |  |  |  |  |
| DBP | 0.986 | 0.966-1.007 | 0.192 |  |  |  |  |
| Heart rate | 0.995 | 0.975-1.016 | 0.660 |  |  |  |  |
| Cardiogenic shock | 2.346 | 0.566-9.714 | 0.298 |  |  |  |  |
| BNP | 1.001 | 1.000-1.001 | **0.003** |  | 1.000 | 0.999-1.001 | 0.412 |
| Creatinine clearance rate | 0.986 | 0.975-0.997 | **0.012** |  | 0.994 | 0.977-1.011 | 0.457 |
| Fasting blood-glucose | 1.060 | 0.976-1.152 | 0.168 |  |  |  |  |
| Triglyceride | 0.905 | 0.716-1.144 | 0.406 |  |  |  |  |
| Total cholesterol | 0.991 | 0.905-1.086 | 0.852 |  |  |  |  |
| LDL-C | 1.008 | 0.732-1.387 | 0.962 |  |  |  |  |
| HDL-C | 0.914 | 0.560-1.493 | 0.720 |  |  |  |  |
| apoA | 0.893 | 0.392-2.031 | 0.787 |  |  |  |  |
| apoB | 1.071 | 0.380-3.018 | 0.897 |  |  |  |  |
| Hcy | 1.005 | 0.995-1.015 | 0.340 |  |  |  |  |
| Lp（a） | 1.000 | 0.999-1.001 | 0.900 |  |  |  |  |
| Albumin | 0.969 | 0.905-1.037 | 0.358 |  |  |  |  |
| Fib | 1.252 | 1.113-1.408 | **<0.001** |  | 1.353 | 1.170-1.564 | **<0.001** |
| D-D | 1.008 | 0.961-1.057 | 0.741 |  |  |  |  |
| PT | 0.993 | 0.941-1.048 | 0.797 |  |  |  |  |
| INR | 0.928 | 0.352-2.448 | 0.880 |  |  |  |  |
| APTT | 0.997 | 0.989-1.005 | 0.471 |  |  |  |  |
| Hemoglobin | 1.004 | 1.000-1.008 | 0.076 |  |  |  |  |
| PLT count | 0.998 | 0.993-1.003 | 0.416 |  |  |  |  |
| WBC count | 0.921 | 0.805-1.055 | 0.236 |  |  |  |  |
| T3 | 0.821 | 0.257-2.619 | 0.739 |  |  |  |  |
| T4 | 0.994 | 0.978-1.009 | 0.433 |  |  |  |  |
| TSH | 1.017 | 1.008-1.026 | **<0.001** |  |  |  |  |
| FT3 | 1.019 | 0.857-1.212 | 0.828 |  |  |  |  |
| FT4 | 1.033 | 1.010-1.057 | **0.005** |  |  |  |  |
| FT3/FT4 | 1.161 | 0.665-2.026 | 0.600 |  |  |  |  |
| TSHI | 1.331 | 1.183-1.498 | **<0.001** |  |  |  |  |
| TT4RI | 1.001 | 1.001-1.002 | **<0.001** |  |  |  |  |
| TFQI | 1.173 | 1.087-1.266 | **<0.001** |  |  |  |  |
| PTFQI | 1.290 | 1.158-1.437 | **<0.001** |  | 1.237 | 1.107-1.383 | **<0.001** |
| LVEF | 0.988 | 0.957-1.020 | 0.452 |  |  |  |  |
| MVD | 2.014 | 1.141-3.556 | **0.016** |  | 1.347 | 0.696-2.609 | 0.377 |
| LM | 0.680 | 0.202-2.293 | 0.534 |  |  |  |  |
| Branches lesions | 1.388 | 0.467-4.125 | 0.555 |  |  |  |  |
| Calcified lesions | 1.446 | 0.673-3.108 | 0.344 |  |  |  |  |
| Thrombosis | 0.424 | 0.098-1.841 | 0.252 |  |  |  |  |
| CTO | 0.919 | 0.397-2.125 | 0.843 |  |  |  |  |
| Long lesion | 1.652 | 0.567-4.819 | 0.358 |  |  |  |  |
| Coronary rotablation | 1.817 | 0.541-6.105 | 0.334 |  |  |  |  |
| Thrombus aspiration | 0.047 | 0.000-84.191 | 0.428 |  |  |  |  |
| IVUS | 1.603 | 0.497-5.174 | 0.430 |  |  |  |  |
| Aspirin | 0.527 | 0.127-2.190 | 0.378 |  |  |  |  |
| Clopidogrel | 0.652 | 0.089-4.777 | 0.674 |  |  |  |  |
| Anticoagulation | 2.092 | 0.503-8.704 | 0.310 |  |  |  |  |
| Statins | 0.526 | 0.127-2.177 | 0.375 |  |  |  |  |
| Diuretics | 2.219 | 1.172-4.202 | **0.014** |  | 1.221 | 0.507-2.942 | 0.656 |
| β-blocker | 0.928 | 0.514-1.673 | 0.803 |  |  |  |  |
| ACEI/ARB | 1.690 | 0.953-3.000 | 0.073 |  |  |  |  |
| Insulin treatment | 2.564 | 1.236-5.231 | **0.011** |  | 1.122 | 0.453-2.775 | 0.804 |

**Table 5 Comparison of baseline characteristics between excluded cases and included cases.**

| **Varies** | **Total**  **（n=1827）** | **Include cases (n=431)** | **Exclude cases (n=1396)** | **P value** |
| --- | --- | --- | --- | --- |
| Male, n (%) | 1301（71.2） | 319 (74.0) | 982（70.3） | 0.160 |
| Age, years | 68（60.76） | 69 (60, 77) | 68（61, 75） | 0.177 |
| Current smoking, n (%) | 638（34.9） | 163 (37.8) | 475（34.0） | 0.243 |
| Alcohol, n (%) | 513（28.1） | 152(35.3) | 361（25.9） | 0.926 |
| Hypertension, n (%) | 1244（68.1） | 299（69.4） | 945（67.6） | 0.838 |
| Diabetes mellitus, n (%) | 546（29.9） | 114（26.5） | 432（30.9） | 0.071 |
| Previous CHD, n (%) | 343（18.8） | 82（19.0） | 261（18.7） | 0.856 |
| Previous PCI, n (%) | 161（8.8） | 30（7.0） | 131（9.4） | 0.121 |
| Previous COPD, n (%) | 57（3.2） | 16（3.7） | 41（2.9） | 0.346 |
| Atrial fibrillation, n (%) | 64（3.5） | 14（3.2） | 50（3.6） | 0.881 |
| Stroke, n (%) | 86（4.7） | 23（5.3） | 63（4.5） | 0.516 |
| Chronic kidney diseases, n(%) | 50（2.7） | 8（1.9） | 42（3.0） | 0.238 |
